# Synthesis and Electrochemical Performance of Graphene @ Halloysite Nanotubes/Sulfur Composites Cathode Materials for Lithium-Sulfur Batteries

**DOI:** 10.3390/ma13225158

**Published:** 2020-11-16

**Authors:** Tian Cen, Yong Zhang, Yanhong Tian, Xuejun Zhang

**Affiliations:** Key Laboratory of Carbon Fiber and Functional Polymers, Ministry of Education, Beijing University of Chemical Technology, Beijing 100029, China; ct070707@163.com (T.C.); zhangyong_smile@126.com (Y.Z.); Zhangxj@mail.buct.edu.cn (X.Z.)

**Keywords:** lithium sulfur battery, sulfur cathode, halloysite nanotubes, acid treatment, reduced graphene oxide

## Abstract

Natural halloysite nanotubes (HNTs) and reduced graphene oxide (RGO) were introduced into the S cathode material to form HNTs/S and RGO@HNTs/S composite electrode to improve the electrochemical performance of Li-S batteries. The effect of acid etching temperature on the morphology and pore structure of HNTs was explored and the morphological characteristics and electrochemical performance of composite electrodes formed by HNTs that after treatment with different acid etching temperatures and RGO were compared. The result shows that the cycling stability and the utilization rate of active substances of the Li-S battery were greatly improved because the pore structure and surface polarity functional groups of HNTs and the introduction of RGO provide a conductive network for insulating sulfur particles. The RGO@HNTs treated by acid treatment at 80 °C (RGO@HNTs-80/S) composite electrode at 0.1 C has an initial capacity of 1134 mAh g^−1^, the discharge capacity after 50 cycles retains 20.1% higher than the normal S electrode and maintains a specific discharge capacity of 556 mAh g^−1^ at 1 C. Therefore, RGO and HNTs can effectively improve the initial discharge specific capacity, cycle performance and rate performance of Li-S batteries.

## 1. Introduction

With the increasing consumption of fossil fuels, air pollution and global warming are becoming more and more serious. Therefore, there has been a steady increase in demand for clean, sustainable, and efficient energy storage devices. Due to the sulfur cathode has a theoretical capacity of 1675 mAh g^−1^ [1]. Thus, Li-S batteries can reach unparalleled gravimetric and volumetric energy densities of 2500 Wh kg^−1^ and 2800 Wh L^−1^ [2], respectively. Lithium-sulfur (Li-S) batteries are becoming one of the most promising candidates in the future. However, limited by the high solubility of intermediate polysulfide in electrolyte, low conductivity of electrode active material and large volume expansion of sulfur during discharge, lithium-sulfur battery is difficult to be applied to practical production [3,4,5].

To address these issues, various carbon materials such as graphene [6], carbon nanotubes [7] and porous carbon [8] have been used to accommodate S cathode to improve the electrical conductivity and reduce the dissolution of intermediate polysulfide. However, a fast-responding sulfur battery requires facile transport of electrolyte/Li^+^ into and out of the sulfur electrode, eventually some hydrophilic soluble polysulfide will diffuse out from the hydrophobic pores due to non-polar carbon not to bind the polar polysulfide, which initiates the shuttle phenomenon [9,10]. Therefore, different metal oxides such as TiO_2_ [11], MnO_2_ [12], Al_2_O_3_ [13], MnO_2_ [14] and macromolecules such as polyethylene glycol (PEG) [15,16] and poly-pyrrole (PPY) [17,18], which were combined into the sulfur electrode to utilize the chemical interaction between oxygen-containing functional groups and polysulfide effectively restrain the shuttle effect in the process of Li-S batteries.

Herein, we introduce polar halloysite nanotubes (HNTs) as cathode hosts in order to improve capacity and cycle performance of Li-S cells. HNTs are aluminosilicate clay mineral with natural hollow nanotubular structure that contains two types of hydroxyl groups, Si-OH groups at the outer surface and Al-OH groups at the inner surface, revealing the positive and negative charges at the inner and outer surface of the HNTs [19,20]. Due to the oppositely charged surface of HNT, the dissociation of lithium salt is promoted by the ordered 3D channels in the electrolyte. Thus, the conductivity of lithium ionic is greatly increased [21]. Because of its structural features, HNTshave been studied for molecular adsorption, molecular encapsulation, storage, transport, and catalysts [22,23,24]. Nanotube format of HNTs can not only just disperse and limit sulfur nanoparticles, but also inhibit the dissolution and migration of polysulfide in the liquid electrolyte solution during the electrochemical reaction process of lithium sulfur battery [25]. The mesoporous structure in the composite electrode of lithium-sulfur batteries can effectively improve the sulfur load of cathode material. Liu [26] used pyrrole as a carbon precursor and HNTs as the templating agent. Mesoporous carbon (MC) was prepared by template etching and combined with sulfur as a composite cathode for lithium-sulfur battery. Due to the large surface area and abundant mesoporous structure, the composite cathode has good cycling stability and rate performance. Thus, the mesoporous structure generated by acid etching on nanotube format of HNTs can improve the sulfur contents and the adsorption of polysulfide in the composite cathode material, which improve the electrochemical performance of lithium-sulfur battery. Although natural HNTs can effectively reduce the shuttle effect of lithium-sulfur batteries, the issue of insulation will degrade the electrochemical performance. Conducting carbon was deposited on the surface of HNT through PDA coating and pyrolysis. HNT@C composites were successfully synthesized and the electrical conductivity of HNT host was thus significantly enhanced, which can effectively reduce the insulation problem of HNT [27]. Due to the high specific surface (theoretical specific surface up to 2630 m^2^ g^−1^), great electrical conductivity (electronic mobility of 200,000 cm^2^ v^−1^ s^−1^), high mechanical strength, good chemical stability and good flexibility, the graphene oxide (GO) has attracted extensive attention from researchers of Li-S battery [28]. Therefore, the electrochemical performance of Li-S batteries can be effectively improved through the mixing of GO, which can effectively improve the conductivity of cathode materials and the volume expansion generated by electrode reaction and prevent the accumulation of nanoparticles [29,30,31].

In this study, HNTs internal and external surfaces were etched with acid to improve surface activity, BET surface and pore volume. Investigating the influence of sulfuric acid treatment temperature on the HNTs morphology and pore structure and comparing the influence of S cathode materials, HNTs/S and RGO@HNTs/S composite electrode on the electrochemical performance of Li-S batteries.

## 2. Experimental

### 2.1. Materials

Natural HNTs was purchased from Hebei Qinglong Mineral Products Co, Qinghaungdao, China. Sulfuric acid (H_2_SO_4_, analytical reagent), Sodium Thiosulfate (Na_2_S_2_O_3_, analytical reagent) and Hydrochloric acid (HCl, analytical reagent) were purchased from Beijing Chemical Works (Beijing, China). Polyvinylpyrrolidone (PVP, analytical reagent) was provided by Beijing Tongguang fine chemical Co (Beijing, China), China. Sulfur (S, analytical reagent) was provided by Sinopharm Chemical Reagent Co, Ltd., Beijing, China.

### 2.2. Acid Treatment of HNTs

HNTs (1 g) were added into H_2_SO_4_ (100 mL, 2 M) solutions. The mixture was magnetically stirred in a water bath for 6 h at various temperatures (70 °C, 80 °C and 90 °C). The product was washed 5 times with deionized water and separated centrifugally [32]. Then sample was dried in the vacuum at 80 °C for 12 h and grounded into powder in an agate mortar. The samples were named HNTs-70, HNTs-80 and HNTs-90 according to the acid treatment temperature and the untreated natural HNTs was named HNTs-0.

### 2.3. Synthesis of HNTs/S Composite

The preparation reaction formula is as follows:

S_2_O_3_^2−^ + 2H^+^ = S + SO_2_↑ + H_2_O

First, HNTs (0.15 g) were added into an aqueous solution of Na_2_S_2_O_3_ (300 mL, 0.04 M) with the presence of Polyvinylpyrrolidone (PVP, Mw~40,000, 0.02 wt.%). The amphiphilic polymer polyvinyl pyrrolidone PVP has a special structure and it is a typical surfactant. In the synthesis of composite electrode materials, the surface modification of HNTs with PVP does not damage the structure of HNTs, but also overcomes the characteristics of surface inertia and easy agglomeration, which can effectively improve the dispersibility of HNTs [33,34]. Then HCl (2.4 mL, 10 M) was added to the above aqueous solution [26]. After reaction for 3 h, the HNTs/S composites were washed with ethanol and water, separated centrifugally, and dried under vacuum for 12 h.

### 2.4. Synthesis of RGO@HNTs/S Composite

First, HNTs (0.085 g) were added into an aqueous solution of Na_2_S_2_O_3_ (300 mL, 0.04 M) with the presence of Polyvinylpyrrolidone (PVP, Mw~40,000, 0.02 wt.%). Then HCl (2.4 mL, 10 M) was added to the above aqueous solution. After reaction for 2 h, adding the RGO (80 mL, 1 mg/mL) solution to react for 1 h [28,35]. At last, the temperature was raised to 90 °C and hydrazine hydrate (1 mL, 0.026 mmol) was added for 3 h. RGO@HNTs/S composites were washed with ethanol and water, separated centrifugally, and dried under vacuum for 12 h.

### 2.5. Materials Characterization

Powder X-ray diffraction (Bruker D8 ADVANCE, Brock, Germany) using Cu Kα radiation at room temperature at 40 kV and 40 mA. Data was collected with the 2θ range of 5–80° at a step size of 0.02°. The morphology of the composite material was analyzed by transmission electron microscope (TEM Hitachi H-800, Tokyo, Japan) and the energy dispersion spectrum of the composite material was analyzed by scanning electron microscope (SEM, S4700, Tokyo, Japan) respectively. Thermos-gravimetric (TG) analyses were conducted using the thermos-gravimetric analyzer (TG 209 F3 Tarsus^®^, Selb, Germany) in nitrogen at a scan rate of 10 °C·min^−1^ from room temperature to 600 °C. The N_2_ adsorption-desorption analysis test was carried out by Micromeritics ASAP 2020 instrument (Micromeritics Instrument Co., Norcross, GA, USA).

### 2.6. Electrochemistry Measurements

The cathodes production process: Firstly, weigh a sulfur-containing composite material, a conductive agent (Super P) and a binder (PVDF) according to a mass ratio of 7:2:1. Then uniformly mixing the sulfur-containing composite material and the conductive agent in a mortar, add the mixed sample into NMP dissolved with PVDF, continuously grinding for 30 min to obtain viscous and uniform cathode slurry. Then the slurry was coated on the circular foam nickel with the diameter of 10mm. Then dry the electrode plate in a vacuum oven (Shanghai Senxin Experimental Instrument Co. Ltd, Shanghai, China) at 60 °C for 12 h, scrape off the slurry on the outer surface of the foam nickel by a scraper. The electrode plate was flatten under a tablet press (Tianjin Keqi Hi-tech Co. Ltd., Tianjin, China) and weighed in final. 2025-type coin cells were assembled in an argon filled glove box (Nanjing Chishun Technology Development Co. Ltd., Nanjing, China) using lithium foil as the anode. The electrolyte was LiClO_4_ (1 M) in 1,3-dioxolane (DOL) and 1,2-dimethoxyethane (DME) (volume ratio 1:1) containing LiNO_3_ (0.15 M). The galvanostatic charge-discharge performance of the cells was tested with LAND CT-2001A instrument (Wuhan, China) and potential window was controlled between 1.7 and 2.8 V at room temperature, the measured capacity was in accordance with the active sulfur content in the cathode materials. Cyclic voltammetry (CV) measurements were carried out on an electrochemistry working station (Potientiostat/Galvanostat Model 263A, Princeton Applied Research, Princeton, NJ, USA) using a voltage range from 1.5 to 3.0 V vs. Li/Li^+^ at a scan rate of 0.1 mV/s. The electrochemical impedance spectroscopy (EIS) data of the cells were measured at room temperature by an electrochemistry working station (Potientiostat/Galvanostat Model 263A, Princeton Applied Research, Princeton, NJ, USA), in the frequency ranging from 100 kHz to 10 mHz by imposing an alternate voltage with an amplitude of 10 mV on the electrode.

## 3. Results and Discussion

**Figure 1:**
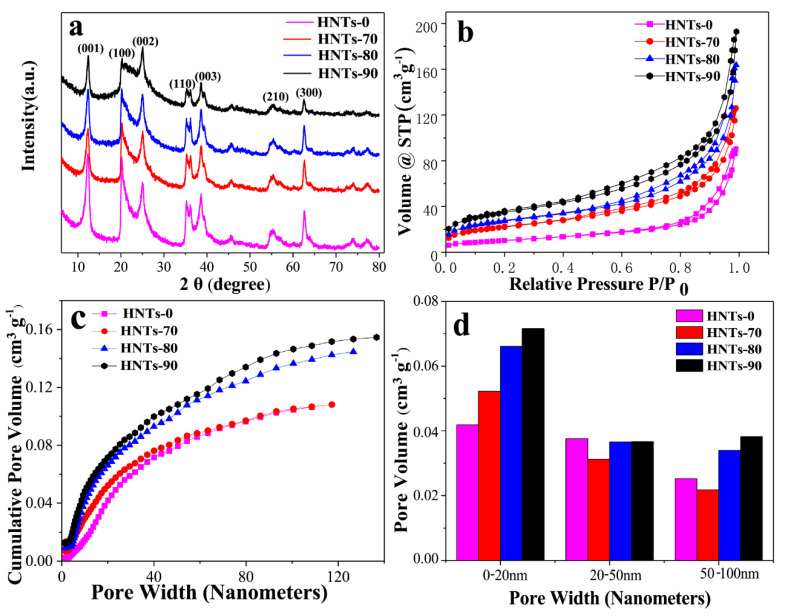
(**a**) X-ray diffraction patterns; (**b**) Nitrogen adsorption/desorption isotherms; (**c**) Cumulative Pore Volume vs Pore Width Curves and (**d**) Pore size distribution histogram of HNTs with different etching temperature (70 °C, 80 °C, 90 °C).

Figure 1a shows the XRD patterns of HNTs at different acid etching temperatures. According to Bragg equation, d (001) = 7.2 Å, the diffraction peaks of HNTs samples treated with different etching temperatures at (001), (100), (002), (110), (003), (210) and (300) coincide with the standard card (JCPDS Card No.09-0453) of 7 Å halloysite, which is the typical diffraction characteristic of 7 Å halloysite. It can be seen that the intensity of diffraction peak decreases with the increase of acid etching temperature in all samples, but the characteristic peak decline degree of HNTs under different acid etching temperature treatment is different. This indicates that the crystal surface of HNTs changed and its structure was destroyed after acid etching. However, with the increase of acid treatment temperature, the damage to the HNTs structure increased and the perforated structure of the tube wall became more pronounced. With the increase of acid etching temperature, the strength of characteristic peak (002) decreased not obviously which means that although acid etching of HNTs can produce pore structure, the crystalline phase is not damaged. Figure 1b shows the N_2_ adsorption/desorption isotherms of HNTs at different etching temperature (70 °C, 80 °C, 90 °C). All the isotherms show the Type IV isotherm [36] according to the basis of IUPAC recommendations, indicating the existence of a mesoporous structures. These mesoporous could inhibit the dissolution of sulfur for cycle. Figure 1c shows cumulative pore volume and pore width cures of natural HNTs and acid treated HNTs. Due to reaction with acid, the volume of pores less than 20 nm was increased with acid treatment temperature. When pore width is under 50 nm, cumulative pore volume distribution curves of HNTs-80 and HNTs-90 coincide basically. It reveals that microporous and mesoporous structure are similar. However, when pore width is beyond 50 nm, two cures gradually split owing to the increase of macroporous pore. Which are intuitively showed in Figure 1d. It is worth noting that pore volume with pore size from 20 to 100 nm of halloysite tubes etched at 70 °C was reduced, which may be ascribed to amorphous silica blocking of a portion of mesoporous and macroporous pore. As a result, the volume of pores less than 20 nm and beyond than 50 nm increases with acid treating temperature and mesoporous with pore size from 20 to 50 nm changes slightly. The BET and cumulative pore volume of samples shown as Figure S1, respectively.

**Figure 2:**
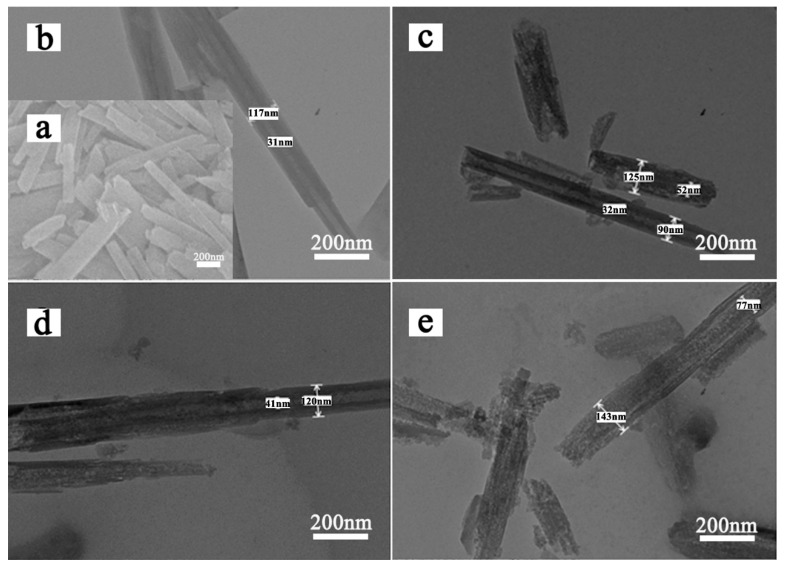
SEM (**a**) images of HNTs-0; TEM images of (**b**) HNTs-0, (**c**) HNTs-70, (**d**) HNTs-80 and (**e**) HNTs-90.

SEM and TEM images of HNTs-0 and acid treated HNTs are shown in Figure 2, exhibiting the morphology and size of the samples. As shown in Figure 2a,b, natural HNTs have obvious hollow tubular structure and average length of 0.6–1.5 µm, with an external diameter in the range of 80–150 nm and an internal diameter of 10–40 nm. Figure 2c–e exhibit the external diameters of HNTs remained unchanged and hollow tubular structure is preserved after etching. However, defects start to develop on the inner and outer with the increase of etching temperature. Because acid can react with HNTs from outer and inner surface, the tube walls of acid treated HNTs exhibit porous structure. This structure will improve the transfer efficiency of Li ions during the cycle because it can reduce the ionic and electronic conduction distance.

Table 1 shows the X-ray photoelectron spectroscopy analysis results of HNTs surface element content before and after acid treatment. It can be seen from Table 1 that the content of Al element and the molar ratio of O/Si in HNTs gradually decrease as the etch temperature rises from 70 °C. When the etching temperature reaches 90 °C, the molar ratio of O/Si is 2.144, which is close to the 2:1 ratio in SiO_2_. Indicating that the oxygen-bearing layer structure of HNTs-90 tube wall structure is greatly destroyed, leading to a large number of reduction of Al-OH and Al-O functional groups. The HNTs tube wall is mainly composed of Si-O functional groups. Therefore, the increase of etching temperature will destroy the HNTs tube structure. The core spectra of XPS analysis in the Supplementary Information as shown in Figures S2 and S3.

**Figure 3:**
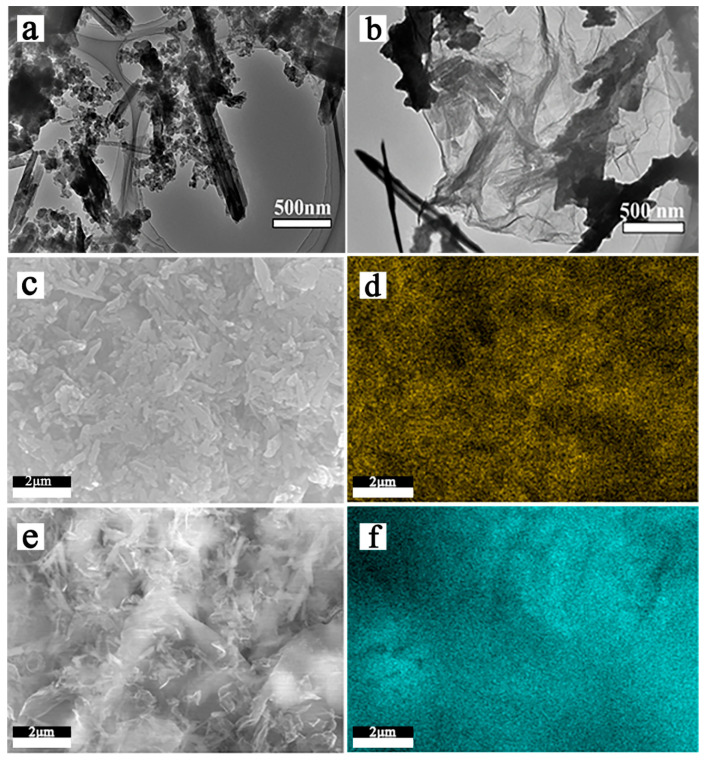
TEM images of HNTs-80/S (**a**) and RGO@HNTs/S composites (**b**); SEM images of HNT-80/S composites (**c**) and sulfur elemental mapping (**d**); SEM images of RGO@HNT-80/S composites (**e**) and sulfur elemental mapping (**f**).

The above results show that the optimal acid etching temperature of HNTs is 70–90 °C. Because of the excellent electrical conductivity, higher specific surface and better structural flexibility of graphene. The HNTs of acid etching was combined with sulfur and graphene to prepare the composite electrode materials. TEM photographs and EDS results of the composite electrode materialswere shown in Figure 3.

Figure 3a,b are TEM images of HNTs-80/S and RGO@HNTs-80/S composite material. It can be observed from the Figure 3a that sulfur particles are uniformly distributed on the HNTs tube walls and in the lumen. The Figure 3b shows that the size of RGO@HNTs-80/S composite material is about 2 μm, which is covered on HNTs-80 by flexible RGO, forming a layer of RGO conductive network structure between the insulating HNTs-80, while sulfur is evenly dispersed on the surface of the folded RGO. Figure 3c,d are SEM images of HNTs-80/S composite material and the sulfur distribution diagram of EDS. It can be clearly seen that sulfur is uniformly dispersed in the HNTs tube wall or lumen, which is conducive to HNTs to better adsorb occurring anionic polysulfide species during charging/discharging and therefore might reduce the shuttle effect. Figure 3e,f can intuitively observe the sulfur distribution in RGO@HNTs-80/S composite materials. There is no obvious sulfur accumulation on the surface of RGO, EDS clearly show that sulfur is more evenly distributed on the RGO@HNTs than HNTs, which is beneficial to increase the charge transfer rate and decrease the electrode polarization in the electrochemical reaction process. The EDX spectrum of HNTs-80/S, RGO@S and RGO@HNTs-80/S shown as Figure S4.

**Figure 4:**
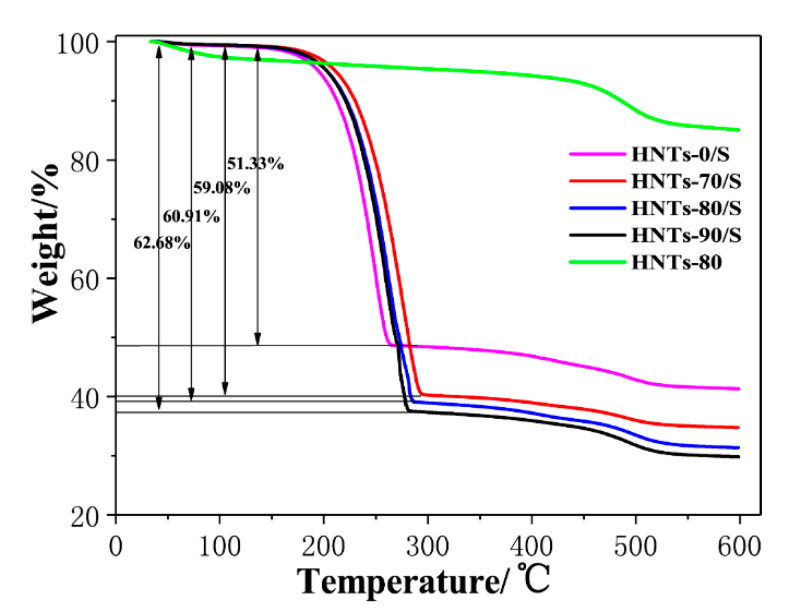
The TG curves of HNTs-80 and HNTs/S composites with different etching temperature.

Figure 4 shows the TG curves of HNTs/S composites at different etching temperatures. It can be seen that the temperature ranges from 150 °C to 300 °C show obvious weight loss, which is caused by the thermal decomposition of sulfur in HNTs/S composite materials [35]. The mass attenuation of HNTs/S, HNTs-70/S, HNTs-80/S, and HNTs-90/S in the temperature range of 150–300 °C is 51.33%, 59.08%, 60.91%, and 62.68% respectively. It indicates that HNTs-90/S contains the most sulfur, followed by HNTs-80/S, HNTs-70/S and HNTs/S. This is because the acid etched HNTs tube has more mesoporous structure, which is easy to absorb sulfur particles. With the increase of etching temperature, the more mesoporous structure, the more sulfur content adsorbed. However, the weight loss of each sample within the range of 450–550 °C is caused by the -OH dehydration of HTNs tube wall [37].

**Figure 5:**
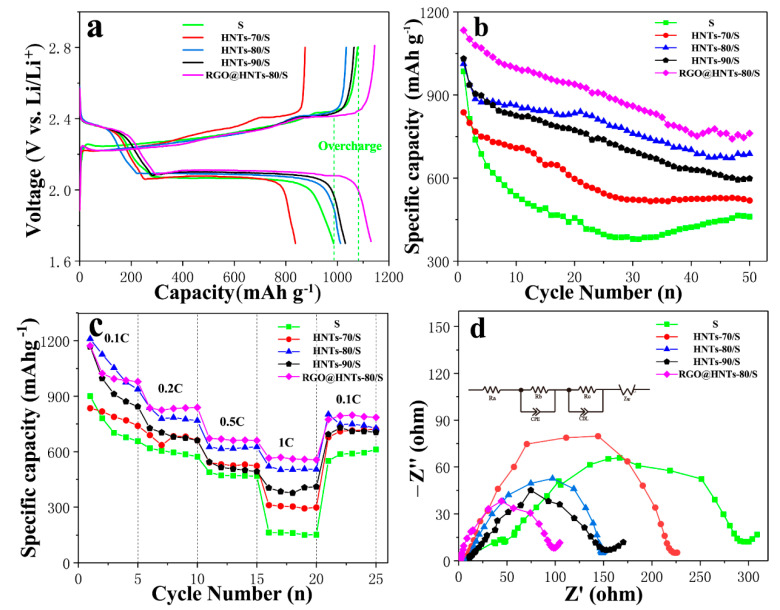
(**a**) Initial galvanostatic discharge/charge voltage profiles; (**b**) Cycling performance comparisons between normal S,HNTs/S and RGO@HNTs/S cathode at current rate of 0.1 C (1 C = 1675 mAh/g); (**c**) Rate capability of normal S, HNTs/S and RGO@HNTs/S cathode with different current densities; (**d**) Impedance spectroscopy analysis of normal S, HNTs/S and RGO@HNTs/S cathode.

Figure 5a shows the initial charge/discharge curves for HNTs/S with different acid treatment temperature and normal S cathode in the potential range of 1.7–2.8 V. There are two typical discharge reaction plateaus observed for the cathode, at ca. 2.4 V and 2.1 V respectively. The initial charge/discharge capacity of normal S cathode, HNTs-70/S, HNTs-80/S, HNTs-90/S and RGO@HNTs-80/S composite electrode was 985.4 mAh g^−1^, 837.1 mAh g^−1^, 1012.5 mAh g^−1^, 1030.7 mAh g^−1^ and 1134 mAh g^−1^ at 0.1 C (1 C = 1675 mAh g^−1^), respectively. Compared with the normal S cathode, HNTs-90/S and HNTs-80/S composite electrodes have higher initial discharge specific capacity, while HNTs-70/S is lower. This may be due to the fact that HNTs-90 and HNTs-80 tube walls have higher porosity than HNTs-70 tube walls, which can be used as a fast transport channel for lithium ions and improve the conductivity of cathode materials. More mesoporous structure can also be adsorbed more active substance sulfur, which has a great influence on the increase of initial discharge specific capacity of electrode materials. Blending the RGO into HNTs-80/S composite material, its initial capacity has been improved. This is because RGO can improve a good conductive network for insulating sulfur particles. In addition, the mesoporous structure of HNTs, which is provides a fast and efficient ion or electron transport channel for electrode materials during charging and discharging. At the same time, it can be clearly seen that the normal S cathode shows an obvious overcharge phenomenon [38], while the introduction of HNTs and RGO@HNTs into the S cathode materials can eliminate the overcharge phenomenon, indicating that the introduction of polar HNTs can greatly inhibit the shuttle effect.

It can be observed in Figure 5b that after 50 cycles, the capacity retention rates of the normal S cathode, HNTs-70/S, HNTs-80/S, HNTs-90/S, and the RGO@HNTS-80/S composite electrode are 47%, 62%, 68%, 58% and 67.1%, respectively. The introduction of HNT can significantly improve the cycling performance of the cathode materials and HNTs-80/S and RGO@HNTs-80/S have relatively high cyclic stability. The effect of HNTs-90/S on the cycle stability is slightly less than that of HNTs-80/S and HNTs-70/S. It is because the solubility of the short chain polysulfide Li_2_S_x_ (x ≤ 4) in the electrolyte is much smaller than that of the long chain polysulfide Li_2_S_x_ (4 < x ≤ 8). The sulfur loading in the micropores can effectively avoid the formation of long-chain polysulfide Li_2_S_x_ (4 < x ≤ 8) that can generates the shuttle effect during the process of charge and discharge. Therefore, the micropores can effectively reduce the shuttle effect caused by the dissolution of long-chain polysulfide Li_2_S_x_ (4 < x ≤ 8) [39].

Figure 5c depicts the electrochemical performance of the composite cathodes at different rates. The cathode was first cycled at 0.1 C, then charge/discharge rate was increased to 0.2 C, 0.5 C, 1 C and last returns to 0.1 C. Compared with S cathode, the HNTs/S composite electrode has better rate performance. This may be attributed to two reasons, as follows: first, the HNTs provides abundant hydroxyl groups on its surface, the wettability between the electrodes and electrolyte is improved. Second, HNTs have strong absorption to polysulphides, which reduce S redistribution and prevent deposition of sulfur on the electrode. Consequently, the HNTs/S cathode has a better rate capability. While the RGO@ HNTs-80/S composite electrode has better rate performance than HNTs-80/S cathode materials. This is because the excellent conductivity of RGO can effectively improve the charge transmission rate for charging and discharging, thus improving the conductivity of electrode materials and accelerating the electrochemical reaction process.

Figure 5d shows the test results of electrochemical impedance spectroscopy (EIS) measurements without cycle. The equivalent circuit as shown in Figure 5d: Ra is the internal resistance of the cell, Rb is the contact resistance and Rc is the diffusion resistance. The Nyquist plots should be composed of a small intercept at high frequency, two semicircles at medium and high frequency and a straight line at low frequency. The intercept with the axis is the internal resistance (Ra) of the battery; the contact resistance (Rb) in the high frequency region is divided into the electrolyte/electrode interface and electrolyte/SEI layer interface and the slope of the straight line in the low frequency region is the diffusion resistance (Rc) [26]. It is exhibiting that the diameter of RGO@HNTs-80/S is the smallest in the high frequency half circle, the diameters of HNTs-80/S and HNTs-90/S are centered, while the diameter of S electrode is largest in the high frequency half circle. It is due to the pore structure on the HNTs-80, HNTs-90 tube wall and the presence of oxygen-containing functional groups, which improve the contact between the electrode and the electrolyte. This can reduce the contact resistance, indicating that the introduction of acid etching HNTs can significantly reduce the internal charge transfer impedance of the battery, thus improving the electrochemical performance of Li-S battery. The introduction of RGO enhances specific transport channels for specific ions and electrons, resulting in a reduced charge transfer impedance, which is also one of the reasons for the improvement of the initial capacity and rate performance of RGO@HNTs-80/S composite electrode.

**Figure 6:**
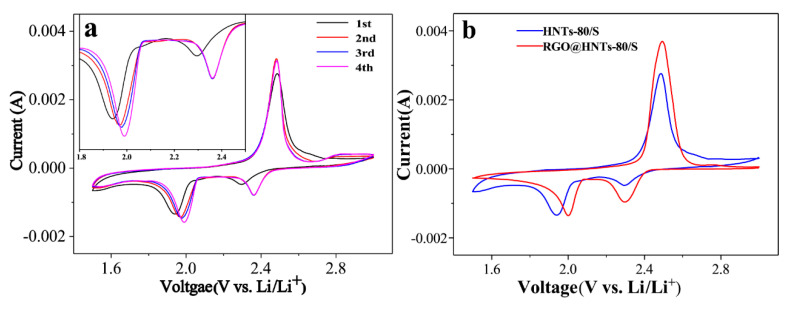
(**a**) Cyclic voltammetry (CV) curves of the HNTs-80/S cathode at 0.1 mV s^−1^ scanning rate. Inset is the enlarged part of CV curves ranging from 1.8 V to 2.5 V; (**b**) Cyclic voltammetry (CV) curves of HNTs-80/S and RGO@HNTs-80/S cathode at 0.1 mV s^−1^ scanning rate.

The cyclic voltammetry curves of the HNTs-80/S composite electrode are shown in Figure 6a. During the first cathode scan, two main reduction peaks at around 2.29 V and 1.94 V (vs. Li^+^/Li) were clearly observed. The peaks at around 2.29 V corresponds to the reduction of S8 to higher-order polysulfides (Li_2_S_x_, 4 ≤ x < 8). The second peak at 1.94 V can be attributed to the further reduction of the higher-order polysulfides to insoluble Li_2_S or Li_2_S_2_ [26]. In the subsequent anodic scan process, a sharp oxidation peaks at 2.49 V were observed and can be attributed to the conversion of Li_2_S and polysulfides into S_8_. By comparing the first cycle, it was found that the reduction peaks of the second and the third cycles shifted to the positive direction, which was because the dissolution of polysulfide would create vacancy in the cathode materials and the introduction of HNTs to increase the pore structure, which are conducive to the infiltration of electrolyte and the transmission of lithium ions, thus the reduction peak shifted to the positive direction [40]. As the number of scans increases, the reduction peak turned to a higher potential, while the oxidation peak transferred to a slightly lower potential. This indicates that after the first activation scan, electrode polarization was weakened and battery reversibility was improved [41,42], which can improve the cycling stability of the battery. Figure 6b compares the cyclic voltammetry curves of the HNTs-80/S and the RGO@HNTs-80/S composite electrode. The reduction peak potential of RGO@HNTs-80/S is higher than HNTs-80/S electrode, indicating that RGO@HNTs-80/S electrode has smaller electrode polarization at high potential, which result in the cathode materials has excellent discharge performance at high current density and obvious improvement of rate performance [21].

**Figure 7:**
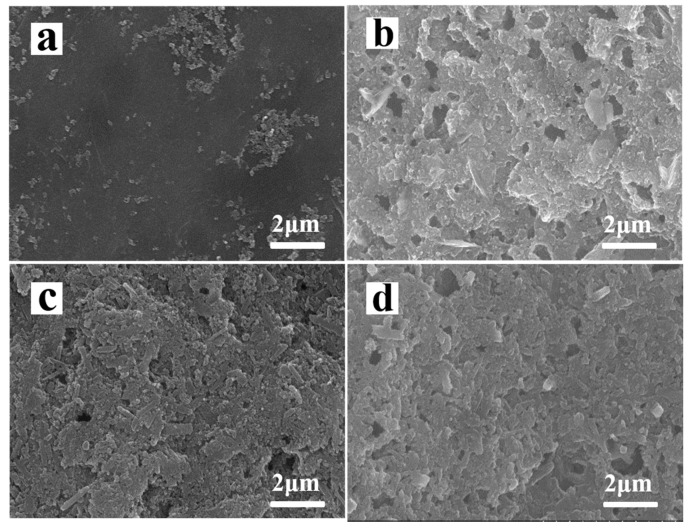
SEM morphologies of cathode: normal sulfur cathode (**a**) before cycling and (**b**) after 50 cycles; HNTs-80/S cathode (**c**) before cycling and (**d**) after 50 cycles.

Figure 7a,b are show the SEM images of normal S electrode before and after 50 cycles. Each cell was cycled for 50 cycles and the electrode was obtained at the end of charge at 2.8 V. A lot of vacancy and serious cracks generated on normal S cathode surface as shown in figure. The vacant area was caused by the loss of active material and the cracks was due to the volume expansion of sulfur upon lithiation. HNTs-80/S electrode has hardly changed after 50 cycles (Figure 5c,d). Moreover, surface morphology of HNTs-80/S electrode is uniform and neat comparison with normal S electrode after 50 cycles. This may be because the pore structure and surface functional groups of HNTs-80 have strong absorption ability for soluble polysulfides and reduce the dissolving and loss of the active materials. The pore structure can provide extra space for volume expansion of sulfur upon lithiation and prevent the electrode from disruption.

## 4. Conclusions

We have studied the effect of acid etching temperature on the morphology and pore structure of HNTs. With the increase of acid treatment temperature, the volume of pores smaller than 20 nm increases and a large number of micropores are formed on the wall of HNTs. With the introduction of RGO, the morphology of RGO@HNTs/S shows a conductive network structure about 2 μm formed by the RGO coating on the insulating HNTs/S. Compared with the normal S electrode, the initial discharge specific capacity, cycle performance and rate performance of the RGO@HNTs/S composite electrode has been greatly improved. The excellent electrochemical performance of the present RGO@HNTs/S composites can be attributed to the pore structure of HNTs and the high electrical conductivity of RGO, which can facilitate good transport of electrons from the poorly conducting sulfur, alleviate the polysulfide shuttle phenomenon and provide fast transport of ions or electrons.

## Figures and Tables

**Table 1:** Surface chemical composition of HNTs with different etching temperature by XPS analysis.

Sample Name	Al (%)	O (%)	Si (%)	O/Si
HNTs-0	16.4	63.4	20.2	3.142
HNTs-70	14.6	63.1	22.3	2.826
HNTs-80	11.7	63.1	25.2	2.508
HNTs-90	7.9	62.8	29.3	2.144

## Supplementary Materials

The following material is available online at https://www.mdpi.com/1996-1944/13/22/5158/s1, Figure S1: BET and cumulative pore volume of HNTs-0, HNTs-70, HNTs-80, HNTs-90, Figure S2: XPS spectra of HNTs-0, HNTs-70, HNTs-80, HNTs-90, Figure S3: High-resolution XPS spectra of Al 2p (a) and Si 2p (b) on HNTs-0, HNTs-70, HNTs-80, HNTs-90, Figure S4: EDX spectrum of the different materials (a) HNTs-80/S; (b) RGO@S; (c) RGO@HNTs-80/S.
