# Supplementary material for: Synthesis and Electrochemical Performance of Graphene @ Halloysite Nanotubes/Sulfur Composites Cathode Materials for Lithium-Sulfur Batteries
Source: Materials (Basel). 2020 Nov 16;13(22):5158. doi: 10.3390/ma13225158 (PMC7720120; doi:10.3390/ma13225158)
Supplement: Supplementary file 1 [file materials-13-05158-s001.pdf]

Synthesis and Electrochemical Performance of Graphene @ Halloysite Nanotubes/Sulfur Composites Cathode Materials for Lithium-Sulfur Batteries

Tian Cen †, Yong Zhang †, Yanhong Tian * and Xuejun Zhang

Key Laboratory of Carbon Fiber and Functional Polymers, Ministry of Education, Beijing University of Chemical Technology, Beijing 100029, China; ct070707@163.com (T.C.); zhangyong_smile@126.com (Y.Z.); Zhangxj@mail.buct.edu.cn (X.Z.)

* Correspondence: Tianyh@mail.buct.edu.cn; Tel.:+86-010-64418679

† Tian Cen and Yong Zhang contributed equally to this work.

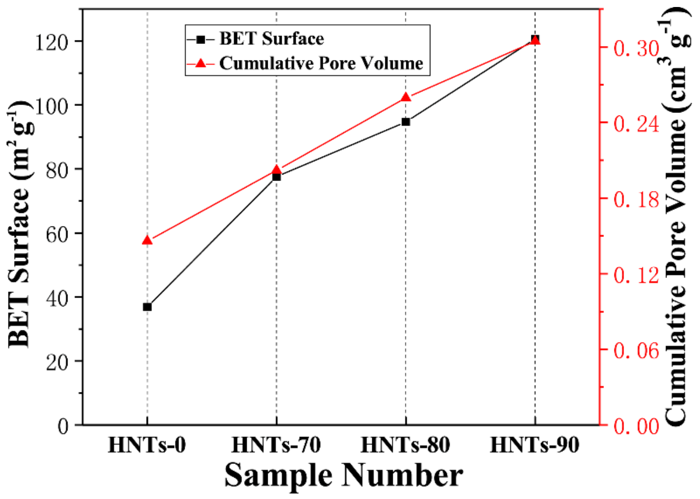

Figure S1. BET and cumulative pore volume of HNTs-0, HNTs-70, HNTs-80, HNTs-90.

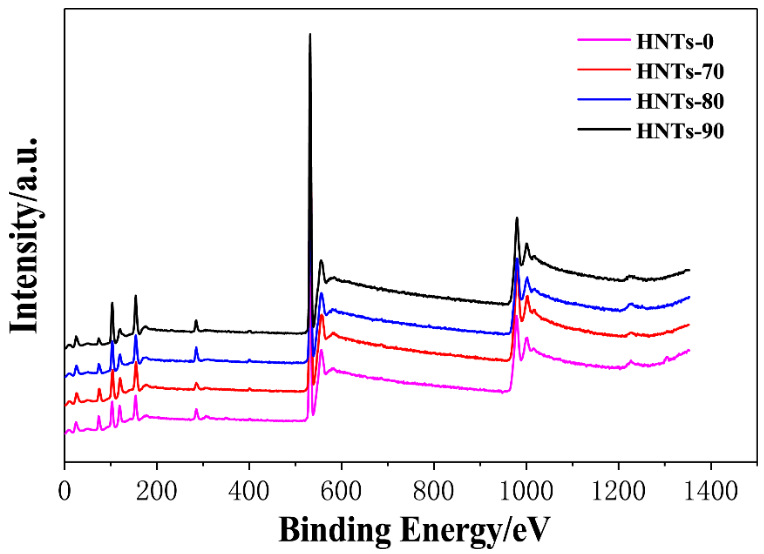

Figure S2. XPS spectra of HNTs-0, HNTs-70, HNTs-80, HNTs-90.

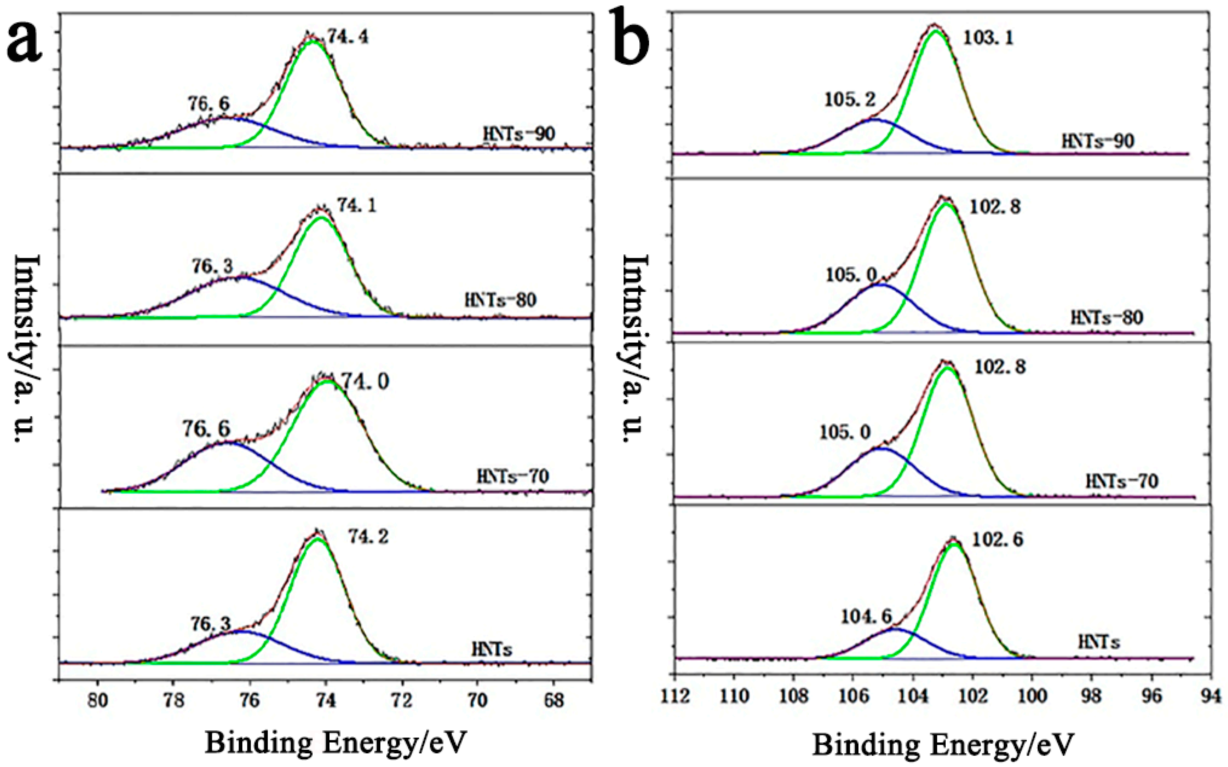

Figure S3. High-resolution XPS spectra of Al 2p (a) and Si 2p (b) on HNTs-0, HNTs-70, HNTs-80, HNTs-90.

The Al 2P peak on the HNTs surface can be fitted to 76.3 eV and 74.2 eV, which belong to Al-OH and Al-O links respectively. While the Si 2P peak on the HNTs surface can be fitted to the groups 102.6 eV and 104.6 eV, respectively. The first belongs to s Si-OH and the second to Si-O bonds. It can be seen that the -OH group increases obviously after acid etching.

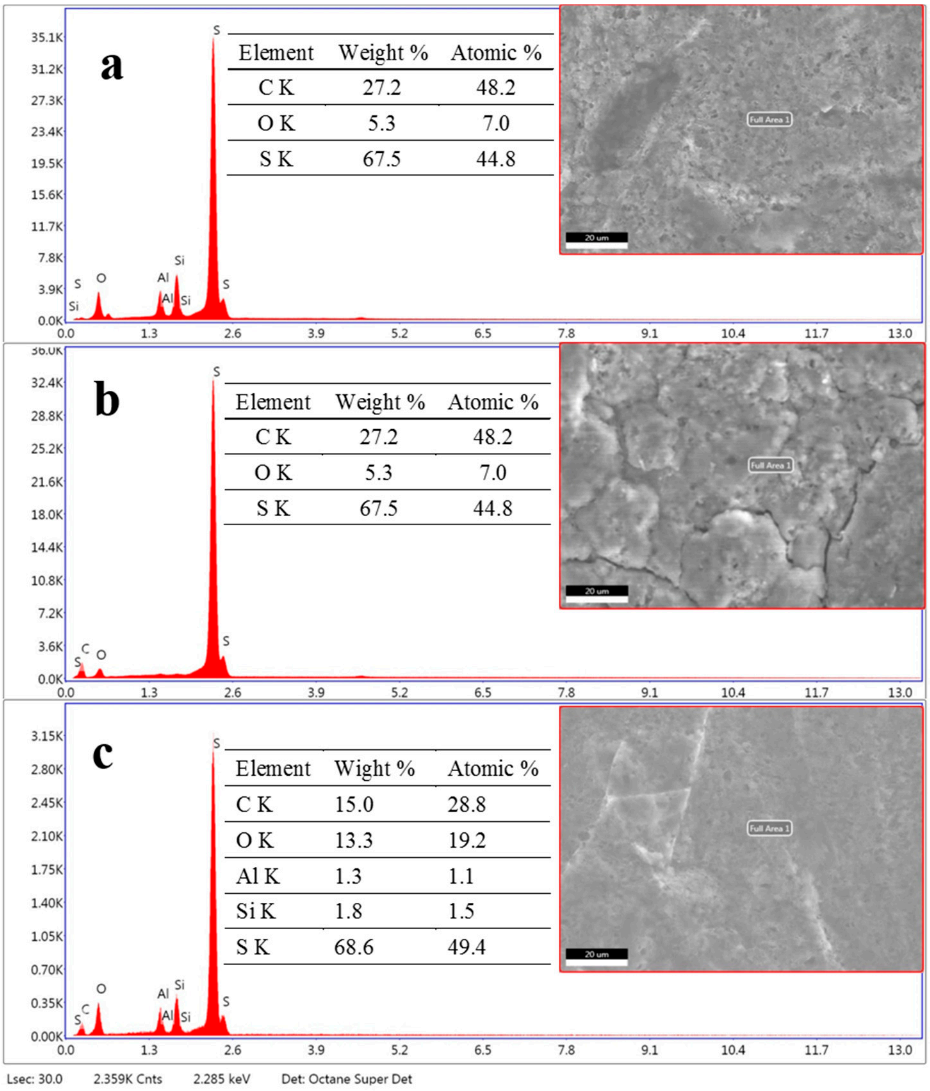

Figure S4. EDX spectrum of the different materials (a) HNTs-80/S; (b) RGO@S; (c) RGO@HNTs-80/S.
